# Defective Mitophagy Impairs Response to Inflammatory Activation of Macrophage-Like Cells

**DOI:** 10.2174/0109298673294643240228105957

**Published:** 2024-03-01

**Authors:** Alexander Nikolaevich Orekhov, Alexander Dmitrievich Zhuravlev, Andrey Yurievich Vinokurov, Nikita Gennadievich Nikiforov, Andrey Vladimirovich Omelchenko, Vasily Nikolaevich Sukhorukov, Vasily Vladimirovich Sinyov, Igor Alexandrovich Sobenin

**Affiliations:** 1 Laboratory of Angiopatalogy, Institute of General Pathology and Pathophysiology, 8 Baltiyskaya Street, 125315, Moscow, Russia;; 2 Cell Physiology & Pathology Laboratory of R&D Center of Biomedical Photonics, Orel State University, 95 Komsomolskaya Street, 30026, Orel, Russia

**Keywords:** Mitochondria, atherosclerosis, defective mitophagy, cybrids, cytokines, chronification of inflammation

## Abstract

**Background and Aims:**

The role of mitophagy in atherosclerosis has been extensively studied during the last few years. It was shown that mitophagy is involved in the regulation of macrophages, which are important players as immune cells in atherosclerosis development. In this study, we investigated the relationship between mitophagy and response to inflammatory stimulation of macrophage-like cells. Six cybrid cell lines with normal mitophagy, that is, increasing in response to stimulation, and 7 lines with defective mitophagy not responding to stimulation were obtained. The objective of the study was to compare the nature of the inflammatory response in normal and defective mitophagy in order to elucidate the role of mitophagy defects in inflammation.

**Methods:**

We used cytoplasmic hybrids (cybrids) as cellular models, created using mitochondrial DNA from different atherosclerosis patients. Mitophagy was stimulated by carbonyl cyanide m-chlorophenyl hydrazone (CCCP) and assessed as the degree of colocalization of mitochondria with lysosomes using confocal microscopy. Western blotting methods were used for the determination of proteins involved in the exact mechanism of mitophagy. Experiments with stimulation of mitophagy show a high correlation between these two approaches (microscopy and blotting). The pro-inflammatory response of cybrids was stimulated with bacterial lipopolysaccharide (LPS). The extent of the inflammatory response was assessed by the secretion of cytokines CCL2, IL8, IL6, IL1β, and TNF measured by ELISA.

**Results:**

Basal level of secretion of cytokines CCL2, IL8 and TNF was 1.5-2 times higher in cultures of cybrids with defective mitophagy compared to cells with normal mitophagy. This suggests a persistently elevated inflammatory response in cells with defective mitophagy, even in the absence of an inflammatory stimulus. Such cells in the tissue will constantly recruit other immune cells, which is characteristic of macrophages derived from monocytes circulating in the blood of patients with atherosclerosis. We observed significant differences in the degree and type of response to inflammatory activation in cybrids with defective mitophagy. These differences were not so much quantitative as they were dramatically qualitative. Compared with cells with normal mitophagy, in cells with defective mitophagy, the relative (to basal) secretion of IL8, IL6 and IL1b increased after the second LPS activation. This indicates a possible lack of tolerance to inflammatory activation in cells with defective mitophagy, since typically, re-activation reveals a smaller pro-inflammatory cytokine response, allowing the inflammatory process to resolve. In cells with normal mitophagy, exactly this normal (tolerant) inflammatory reaction was observed.

**Conclusion:**

Data on the involvement of mitophagy, including defective mitophagy, in disturbances of the inflammatory response in sepsis, viral infections, autoimmune diseases and other pathologies have previously been reported. In this work, we studied the role of defective mitophagy in non-infectious chronic inflammatory diseases using the example of atherosclerosis. We showed a dramatic disruption of the inflammatory response associated with defective mitophagy. Compared with cybrids with normal mitophagy, in cybrids with defective mitophagy, the secretion of all studied cytokines changed significantly both quantitatively and qualitatively. In particular, the secretion of 3 of 5 cytokines demonstrated an intolerant inflammatory response manifested by increased secretion after repeated inflammatory stimulation. Such an intolerant reaction likely indicates a significant disruption of the pro-inflammatory response of macrophages, which can contribute to the chronification of inflammation. Elucidating the mechanisms of chronification of inflammation is extremely important for the search for fundamentally new pharmacological targets and the development of drugs for the prevention and treatment of chronic inflammatory diseases, including atherosclerosis and diseases characteristic of inflammation. Such diseases account for up to 80% of morbidity and mortality.

## 1 INTRODUCTION

The emergence and development of atherosclerotic lesions in the arterial wall are closely associated with local chronic inflammation. The inflammatory response is a normal and very useful protective device. It ends with resolution quite quickly, but if its regulation is disrupted, acute inflammation can become chronic. The mechanisms of chronification of inflammation are not clear. An important factor in the chronification of the inflammatory response is mitochondrial dysfunction [1]. The last decade has revealed a growing interest in the role of mitochondria in atherosclerosis, and, in particular, in mitophagy, which is a cellular process that involves the selective degradation of damaged or dysfunctional mitochondria *via* autophagy. More than a hundred articles have been published on this topic [2]. Mitophagy is indispensable for the turnover and recycling of the cellular mitochondrial pool, and its deficiency leads to serious consequences for the cell and the whole organism, which is associated with many diseases [3, 4]. Recent research suggests that mitophagy may play a role in atherosclerosis by influencing the health and function of endothelial cells [5, 6]. Furthermore, mitophagy is involved in the regulation of the function of macrophages, which, as immune cells, play a key role in the development of atherosclerosis [7]. The discovery of the mechanism of chronification of inflammation opens up new possibilities not only in the prevention and treatment of atherosclerosis but also in other chronic inflammatory diseases. Such diseases account for up to 80% of morbidity and mortality, so research on this topic is of great medical and social importance. Chronic inflammatory diseases manifest themselves in the form of persistent inflammation that continues for a long time. In this case, the immune system mistakenly triggers an inflammatory reaction non-stop, even in the absence of the original cause of inflammation. In chronic inflammatory diseases, dysregulation of the secretion of inflammatory cytokines is observed. Our main interest is such an inflammatory disease as atherosclerosis, so we do not study types of dysregulations of inflammatory reactions that are uncharacteristic of atherosclerosis, such as cytokine deficiency or cytokine storm. We are looking for mechanisms of chronification of inflammation accompanied by sustained secretion of cytokines. In particular, this occurs with intolerant secretion of cytokines. In normal acute inflammation, macrophages secrete cytokines in response to inflammatory stimuli such as pathogens or endogenous damaged molecules or particles. With repeated inflammatory stimulation, tolerance is observed, manifested in the form of a much lower level of cytokine secretion, which is important for preventing chronic inflammation. However, if the regulation of cytokine secretion is impaired, repeated inflammatory activation can cause the same or even higher levels of secretion, that is, the inflammatory response becomes intolerant. This will inevitably lead to chronification of inflammation. We are looking for the causes of the non-tolerant inflammatory response to explain the mechanisms of chronification of inflammation. To this end, in the present work, we investigated whether there is a relationship between defective mitophagy and intolerant inflammatory response of macrophages. Here, we report our own data indicating that defective mitophagy impairs response to inflammatory activation of macrophage-like cells.

## 2 MATERIALS AND METHODS

### 2.1 Cybrid Lines

The heteroplasmic mutations of the mitochondrial genome associated with atherosclerotic lesions and atherosclerotic diseases have been identified in our previous studies [7-11].

For the creation of cybrid cell cultures, the donors were recruited from the cohort of coronary heart disease patients who had previously passed mtDNA genotyping, and those patients with an isolated predominance of certain mutant mtDNA variants were selected. This study was kept in accordance with the Helsinki Declaration of 1975 as revised in 1983, 2008 and 2013, and was approved by Ethics Committee of the Institute for Atherosclerosis Research (Moscow, Russia); Approval No. 26 of March 10, 2023. Written informed consent was obtained from each donor. We have followed the Helsinki Guidelines for human participants. Briefly, platelets were isolated from whole blood by ficoll-urografin density gradient centrifugation. Then, the cytoplasmic transfer of mtDNA from platelets into amitochondrial ρ0 cells produced from the THP-1 cell line purchased from the American Type Culture Collection (ATCC, USA) has been performed by the PEG fusion method, with cytoplasmic hybrid (cybrid) as a result.

In detail, cybrid lines were obtained using the technique of King and Attardi [12] adopted in application to the THP-1 cell line [11, 13]. The principle of the standard method is based on the use of inhibitors of mtDNA replication by ethidium bromide to obtain mitochondrial-free (ρ0) cells from the original THP-1 cell line. Cells were cultured in RPMI-1640 medium (GIBCO) containing 300 mg/ml L-glutamine 2x10^-5^ M 2-mercaptoethanol, 110 μg/ml sodium pyruvate, 2500 mg/l glucose, 50 U/ml penicillin-streptomycin, 10% fetal bovine serum. The cells were maintained in culture for 18 weeks, with passage every 2-3 days. Next, ethidium bromide was excluded from the growth medium and the resulting cells were cultivated in the growth medium supplemented with 200 μg/mL uridine. To obtain cybrid cultures, the PEG-fusion technique was used, which ensures the penetration of foreign mitochondria into ρ0 cells by forming pores in the cellular cytoplasmic membranes. The use of platelets greatly simplifies the protocol for obtaining cybrid lines, since platelets contain only the mitochondrial genome. Platelets were isolated from the whole blood of donors by centrifugation on a Ficoll-Paque (GE Healthcare, IL, USA) density gradient. Sodium citrate in physiological saline was added to the blood obtained from donor patients in a 1:1 ratio. The resulting mixture was centrifuged for 20 min at 200 g at 12°C. Three-quarters of the supernatant (plasma) was taken and centrifuged for 20 min at 1500 g at 15°C, the supernatant was removed, and 11 ml of physiological saline was added to the remaining sediment. The resulting platelet fraction was stored for cryopreservation after the addition of 15 ml of sterile DMSO and 3 ml of fetal calf serum. Next, cells were subjected to controlled freezing in cryovials (10°C/min) for 8 hours until a temperature of -80°C was reached. The samples were then stored in liquid nitrogen. In this study, we used 13 lines of cybrids and the maternal line THP-1.

### 2.2 Mitophagy

Specific mitochondrial autophagy (mitophagy) leading to sequestration of damaged mitochondria into autophagosomes for subsequent lysosomal degradation can be realized by the different pathways, *i.e.* PINK1/Parkin-independent or mitophagy receptor pathway [14]. Western blotting methods are used for the determination of proteins involved in the exact mechanism of mitophagy, while confocal microscopy using mitochondria and lysosomes-specific fluorescent probes allows us to estimate the total fraction of mitochondria co-localized with lysosomes, regardless of any specific pathway [15, 16]. Experiments with stimulation of mitophagy show a high correlation between these two approaches [17], which can be used independently. Routinely mitophagy was determined by confocal laser scanning microscopy using MitoTracker Green FM and LysoTracker Red DND-99 as the degree of colocalization of mitochondria with the lysosomes after 12-hour incubation of cells without additives or with the addition of 2 μM carbonyl cyanide m-chlorophenyl hydrazone (CCCP). Confocal images were then processed using the Cell Profiler package [18]. Using the software built into Cell Profiler, single cells were first identified, then the average correlation of fluorescence intensities of mitochondria and lysosomes for each cell was quantified, and the average colocalization value was calculated for each cell line according to the recommendations [19].

### 2.3 Pro-Inflammatory Response

To compare the pro-inflammatory response of cybrids, the inflammatory stimulation with bacterial lipopolysaccharide (LPS) was used. LPS (1μg/ml) was added to the cybrid culture for 4 hours (1^st^ stimulation, or “1^st^ hit”). Cells were then washed with PBS, and LPS was added for another 20 hours (2^nd^ hit). This incubation time was chosen for convenient procedure since the generally accepted time to assess the THP-1 inflammatory response (following LPS pre-stimulation) ranges from 16 to 24 hours [20, 21]. Upon completion of the experiment, the secretion of cytokines CCL2, IL8, IL6, IL1b and TNF was measured using ELISA. ELISAs were performed using R&D Systems DuoSet ELISA kits for the corresponding cytokines according to the manufacturer's protocol. The detailed protocol is described elsewhere [22]. The basal secretion before the first stimulation (1^st^ hit) was considered to be the secretion of the cytokine by cells without LPS. The basal secretion prior to the second stimulation (2^nd^ hit) was considered to be the secretion from the cells that had received the first stimulation (1^st^ hit).

### 2.4 Statistical Analysis

The statistical analysis was carried out using the R programming language in the RStudio environment. Primary processing and preparation of data was carried out using specialized packages. For Excel files, the “openxlsx” and “XLConnect” packages were used, as well as the “stringr” package for regular expressions. Data formatting was carried out using the “reshape” and “tidyr” packages. Data visualization was carried out using “ggplot2” package. The analysis of the statistical power of the tests and the effect size was performed using the “pwr” package. Before analyzing the differences in the change in cytokine secretion, a bootstrap procedure was used, which consisted of calculating 10,000 random samples of values and calculating the differences between the relative increase in cytokine secretion during defective and normal mitophagy, as well as after the first and second stimulations. The calculation of 95% confidence intervals (CI) for the differences in the proportions, type I error and type II error was carried out using the basic set of R functions. The *p*-value of less than 0.05 was considered statistically significant. The strength of the difference between secretion in defective and normal mitophagy was inferred from the value of the effect size. With an absolute effect size of up to 0.2, the strength of the difference was considered as small, from 0.3 to 0.8 as medium, and above 0.8 as strong.

## 3 RESULTS

### 3.1 Mitophagy

We have previously characterized the atherosclerosis-associated mitochondrial DNA (mtDNA) mutations (genome variants) in human atherosclerotic lesions and blood cells [8, 9]. To study the role of these mutations in atherogenesis, we created several cybrid cell lines based on macrophage-like THP-1 cells carrying different mtDNA mutations [10]. Thirteen cybrid lines obtained in this way were also used in the present work to reveal the relationship between defective mitophagy and pro-inflammatory response. In 6 of the 13 cybrid lines, mitophagy was activated by the addition of СCCP in a normal way. However, in the remaining 7 cybrid lines, mitophagy was not activated by the addition of CCCP and was considered by us as defective mitophagy (Table **1**).

### 3.2 Inflammatory Response

#### 3.2.1 Basal Secretion of Cytokines

Table **2** presents basal cytokine secretion by unstimulated cells. Noteworthy is the higher secretion of the cytokines CCL2 (C-C motif ligand 2), also known as monocyte chemoattractant protein-1 (MCP-1), and IL8 (interleukin-8). After the 1^st^ hit, and prior to the 2^nd^ hit, basal secretion of all studied cytokines was significantly higher both in cultures with normal and defective mitophagy. Thus, after the 1^st^ hit, the level of cytokine secretion did not return to baseline but remained significantly higher than before the inflammatory stimulation for at least 20 hours. It should be noted that in cultures with defective mitophagy, the basal secretion of cytokines CCL2, IL8 and TNF on both the 1^st^ and the 2^nd^ hits was higher than the secretion of these cytokines in cultures with normal mitophagy; moreover, these differences were statistically significant for these 3 cytokines upon the 2^nd^ hit, and for CCL2 and TNF also upon the 1^st^ hit. It can be concluded that basal secretion of some cytokines is higher in cultures of cybrids with defective mitophagy. This suggests a persistently elevated inflammatory response in cells with defective mitophagy, even in the absence of an inflammatory stimulus. Such cells in the tissue will constantly recruit other immune cells, which is characteristic of macrophages derived from monocytes circulating in the blood of patients with atherosclerosis. We have recently shown “that macrophages derived from LPS stimulated monocytes in individuals with subclinical atherosclerosis exhibited increased secretion of IL6, IL10 and CCL2, which was associated with intima-media thickness” [22]. Increased secretion of cytokines by macrophages may contribute to chronic local inflammation in the vascular wall by recruiting other immune cells. Perhaps it is precisely those cells circulating in human blood that have increased secretion of pro-inflammatory cytokines that have defective mitophagy.

#### 3.2.2 LPS Stimulated Secretion of Cytokines

The secretion of cytokines CCL2 and IL8 after the first stimulation but before the second was, respectively, 3.4 and 1.6 times higher in cultures of cybrids with defective mitophagy compared with cells with normal mitophagy (Table **1**). The secretion of other cytokines did not differ significantly between normal and defective mitophagy.

**Fig. (1):**
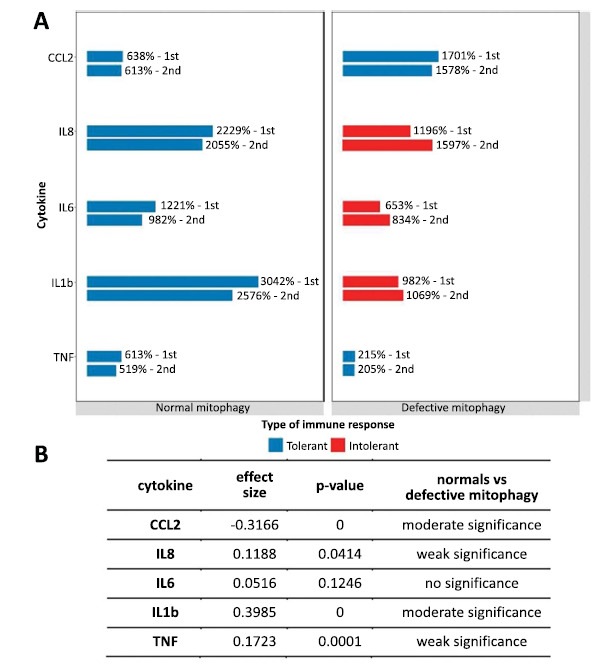
Relative increase in cytokine secretion over basal secretion (100%) after the first (1^st^) and second (2^nd^) LPS stimulation. (**A**) Percentages show an increase in the level of cytokine secretion after the first and second stimulation relative to the level of secretion before the first and second stimulations, taken as 100%, respectively. The red horizontal bars show cytokines with non-tolerant responses. After the second stimulation, slight but statistically significant differences in secretion between the first and second stimulation were revealed for all cytokines (*p*-value = 0). At the same time, for the cytokines IL6, IL8 and IL1b, a higher level of secretion was revealed after the second stimulation compared to the first one. This indicates a lack of tolerance of the response to repeated inflammatory stimulation for these interleukins. (**B**) Comparative analysis of differences in the proportions of cytokine secretion after the first and second stimulation (see Materials and Methods).

When stimulated with LPS, we observed significant differences in the degree and type of response to stimulation in cybrids with defective mitophagy. These differences were not so much quantitative as they were dramatic qualitative. Compared with cells with normal mitophagy, in cells with defective mitophagy, the relative (to basal) secretion of IL8, IL6 and IL1b increased after the second LPS stimulation. This indicates a possible lack of tolerance to inflammatory stimulation in cells with defective mitophagy, since typically, re-stimulation reveals a smaller pro-inflammatory cytokine response, allowing the inflammatory process to resolve. In cells with normal mitophagy, exactly this normal (tolerant) inflammatory reaction was observed (Fig. **1**).

Fig. (**1**) shows the relative increase in cytokine secretion after the first and second activation with LPS. Changes in the levels of cytokine secretion relative to basal secretion (before LPS activation) are shown; levels are expressed as a percentage of basal secretion (100%). This horizontal bar is named “1^st^”. The horizontal bar called “2^nd^” is the level of secretion after re-activation relative to the secretion after the first activation but before LPS re-activation.

To prove the significance of the difference in the relative levels of cytokine secretion between cells with defective and normal mitophagy, a resampling procedure in the form of bootstrap analysis was used. Resampling makes it possible to use more powerful and sensitive statistical tests to examine differences and obtain more accurate statistical estimates of those differences. In this case, the nature and type of distribution of the studied variables do not change and the relationships in the studied parameters are not affected.

Differences in the degree and type of response to stimulation in normal and defective mitophagy were clear. Thus, the secretion of CCL2 both after the first and after the second activation was significantly higher in cells with defective mitophagy. However, secretion after the second stimulation in both normal and defective mitophagy was lower than after the first one. This indicates a tolerant response to inflammatory activation. Secretion of the three cytokines (IL8, IL6 and IL1b) was lower in defective mitophagy. Also, in defective mitophagy after the second activation, the secretion of these three cytokines was higher than after the first one. This indicates a lack of tolerance to inflammatory activation. Although TNF secretion in defective mitophagy was lower than in normal mitophagy, response tolerance was maintained.

## 4 DISCUSSION

The intolerant inflammatory response of macrophages that we studied in this work is a process known as trained immunity or innate (learned) immune memory. The concept of trained immunity has been intensively and extensively developed since 2011 [23]. Over the past decade, it has become clear that not only adaptive immune cells but also various types of immune and non-immune cells of the central and peripheral immune system can develop trained immunity [24]. Trained immunity is characterized by the increased production of proinflammatory cytokines upon a secondary inflammatory activation. This phenomenon is defined as ” an enhanced immune response to reinfection”. Without a doubt, in the struggle for innate immunity to pathogens, this is a very useful tool. The authors of the concept of trained immunity noting the positive role of trained immunity in infections point to its negative participation in the development of various non-infectious pathologies. This leads to misunderstandings as the question unwittingly arises whether trained immunity is a good thing or a bad thing. We believe that trained immunity is not typical in the norm, but in non-infectious diseases, increased secretion of cytokines after re-activation is clearly associated with pathology. To avoid confusion, we use the term “trained immunity” for infections only, and for non-infectious diseases, the term “intolerant response”.

The main objective of our work was to identify the relationship between mitochondria and the intolerant inflammatory response of macrophages. Mitochondria not only play a central role in metabolism but are also a key player in immune and inflammatory reactions [25, 26]. The immunogenic abilities of dysfunctional mitochondria are known [27, 28]. Dysfunctional mitochondria, including those resulting from defective mitophagy, are also associated with impaired immune response [29]. Defective mitophagy causes excessive secretion of proinflammatory cytokines; this, in turn, activates innate immune responses and inflammation at the level of immune cells, which is characteristic of autoimmune diseases [30]. Mitophagy may play a central role in sepsis [31]; suppression of mitophagy by drugs inhibits macrophage migration, which exacerbates inflammation [32]. However, excessive mitophagy is also bad because it can lead to apoptosis of macrophages, which further aggravates the inflammatory response [33]. Viral infection induces mitophagy to inhibit apoptosis and inflammation through NLRP3 [34-37].

In contrast to the pathologies listed above, we studied the role of defective mitophagy in non-infectious chronic inflammatory diseases using the example of atherosclerosis. We showed that the pro-inflammatory response to LPS activation in cultures of cybrids with defective mitophagy differed dramatically from the response in cell lines with normal mitophagy. This finding is in line with our previous assumptions about the important role of defective mitophagy in disrupting the response to inflammatory macrophage activation, which ultimately leads to local chronification of inflammation in the arterial wall [38-40]. In particular, the cytokines IL8, IL6 and IL1β in cells with defective mitophagy showed an intolerant response to repeated inflammatory stimulation. Under normal conditions, after repeated activation, response tolerance occurs, that is, the secretion of pro-inflammatory cytokines is reduced compared to the initial activation, and this contributes to the rapid resolution of inflammation.

In cells with defective mitophagy, as we have shown, there is an increase in the secretion of cytokines IL8, IL6 and IL1β as a result of repeated activation, which obviously should lead to chronic inflammation.

Defective mitophagy was associated with altered secretion of all studied pro-inflammatory cytokines, regardless of their role in the inflammatory response and mechanism of action. For example, CCL2 (C-C motif ligand 2), also known as monocyte chemoattractant protein-1 (MCP-1), and IL8 (interleukin-8) are chemokines, which are a type of cytokine involved in inflammation. While both CCL2 and IL8 play roles in the pro-inflammatory response, their mechanisms of action differ from other cytokines in several ways [41]. CCL2 and IL8 are primarily known for their ability to recruit and attract immune cells, particularly monocytes and neutrophils, respectively, to the site of inflammation. Other cytokines may have proinflammatory effects, but they may not possess the same chemotactic activity as CCL2 and IL8. CCL2 primarily attracts monocytes, which are a type of white blood cell involved in the immune response, while IL8 primarily attracts neutrophils, another type of white blood cell.

IL-6 is a pleiotropic, pro-inflammatory cytokine that plays a critical role in the immune response and inflammation [42]. Its unique properties have made it an attractive target for therapeutic intervention in a variety of diseases. Despite the fact that the level of IL-6 secretion in cells with normal and defective mitophagy is similar, the nature of the effect is fundamentally different. In contrast to normal mitophagy, defective mitophagy is associated with an intolerant IL-6 response.

Unlike most cytokines, IL-1β is synthesized as an inactive precursor (pro-IL-1β) that requires proteolytic processing by caspase-1 to become biologically active [43]. This proteolytic cleavage is triggered by the assembly of a large protein complex called the inflammasome, which is formed in response to various stimuli, including infections, tissue damage, and metabolic stress.

Tumor necrosis factor (TNF) is a cytokine that plays a crucial role in the immune system and inflammation [44]. TNF differs from other cytokines in its structure, source, function, and receptor binding. Its broad range of functions and involvement in various diseases make it a key target for therapeutic intervention.

Cybrids are commonly used to study the role of the mitochondrial genome in normal and pathological cellular responses. In particular, we have recently shown that editing the mitochondrial genome, which involves removing a certain atherosclerosis-associated mitochondrial mutation, leads to the normalization of mitophagy and the appearance of a tolerant inflammatory response in cybrids with initially defective mitophagy and an intolerant reaction [45].

In the present work, we did not investigate the relationship of atherosclerosis-associated mitochondrial mutations in defective mitophagy and/or intolerant response of macrophages to inflammatory activation. This is the subject of our ongoing research and future publications. However, we found that cybrid lines carrying the mitochondrial genome from different patients respond differently to stimulation of mitophagy. Half of the cybrids had normal mitophagy, while the other half had defective mitophagy. We decided to use these differences as a model to study the involvement of defective mitophagy in the formation of an intolerant inflammatory response in macrophages. Despite the fact that the possible existence of a relationship between defective mitophagy and inflammation has been repeatedly pointed out (see above), a direct association has been identified for the first time. This confirmed our hypothesis about the important role of defective mitophagy in the formation of an intolerant inflammatory response and chronic inflammation in general.

In addition to scientific significance, our finding may have practical value if we consider defective mitophagy as a pharmacological target for the development of fundamentally new approaches to the development of anti-inflammatory drugs. There is a lot of serious work ahead in this direction since the mechanisms of formation of defective mitophagy are not known. The most direct way to identify these mechanisms may be based on a comparison of gene regulation during normal and defective mitophagy in cybrids. Identification of signaling and metabolic pathways, as well as key genes and proteins characteristic specifically for defective mitophagy, should help in uncovering the causes of defective mitophagy.

## CONCLUSION

In conclusion, in defective mitophagy, the secretion of all studied cytokines changed significantly, despite considerable differences between them. These changes were both quantitative and qualitative. Undoubtedly, such serious changes indicate a significant disruption of the pro-inflammatory response of macrophages, which can lead, along with other factors, to the chronification of inflammation. Given the medical and social importance and the widespread prevalence of chronic diseases, it can be assumed that defective mitophagy and factors responsible for mitophagy defects can become a fundamentally new pharmacological target for the development of anti-inflammatory therapy.

## Figures and Tables

**Table 1:** Normal and defective mitophagy in cybrid cell lines.

**Cell Line**	**Increase in CCCP-induced Mitophagy Relative to Basal Level**	***p*-value**	**Type of Mitophagy**
THP1	1,32 ± 0,10	< 0.05	normal
TC-522	1,11 ± 0,09	< 0.05	normal
TC521	1,13 ± 0,11	< 0.05	normal
HSM2	1,34 ± 0,11	< 0.05	normal
TCI-521	1,15 ± 0,07	< 0.05	normal
TCN-521	1,18 ± 0,10	< 0.05	normal
TCP-521	1,13 ± 0,10	< 0.05	normal
TC-520	0,71 ± 0,07	< 0.05	defective
TC-HSMAM3	1,05 ± 0,08	> 0.05	defective
TC-HSMAM2	0,96 ± 0,07	> 0.05	defective
TC-HSMAM1	1,02 ± 0,09	> 0.05	defective
TC-LSM2	0,80 ± 0,05	< 0.05	defective
TC-LSM1	0,67 ± 0,07	< 0.05	defective
TC-HSM1	1,02 ± 0,08	> 0.05	defective

**Note:** Data are presented as mean increase in CCCP-induced mitophagy relative to basal level ± S.E. Data combine the results of three independent experiments, and significance was assessed using the Mann-Whittney test.

**Table 2:** Basal secretion of cytokines (pg/ml) in cultures of cybrids with normal and defective mitophagy.

-	**Normal Mitophagy**		-	**Defective Mitophagy**		-		
-	**1^st^ Hit (I)**	**2^nd^ Hit (II)**	***p*, I *vs* II**	**1^st^ hit (III)**	**2^nd^ hit (IV)**	***p*, III *vs* IV**	***p*, I *vs* III**	***p*, II *vs* IV**
**CCL2**	61.7±10.8	104.0±2.8	0.038	89.9±22.1	357.9±15.8	0.0006	0.14	0.003
**IL8**	37.2±8.1	188.0±15.6	0.023	75.6±9.1	307.3±15.4	0.008	0.003	0.02
**IL6**	2.1±0.6	4.6±1.3	0.041	1.8±0.7	3.8±0.7	0.002	0.3	0.5
**IL1b**	1.0±0.5	4.4±0.8	0.037	1.1±0.2	4.0±0.5	0.006	0.8	0.7
**TNF**	14.5±1.5	27.2±1.5	0.039	20.1±1.9	29.8±2.5	0.03	0.01	0.5

**Note:** The mean values and standard deviations of three independent experiments are given. The significance of differences was assessed by a paired t-test after applying the appropriate bootstrap procedure.

## Data Availability

The data and supportive information are available within the article.

## LIST OF ABBREVIATIONS

CCCP: Carbonyl Cyanide m-chlorophenyl Hydrazone
CCL2: C-C Motif Ligand 2
ELISA: Enzyme-linked Immunosorbent Assay
IL: Interleukin
LPS: Lipopolysaccharide
MCP-1: Monocyte Chemoattractant Protein-1
NLRP3: Nucleotide-binding Domain Leucine-rich Repeat Containing Family Pyrin Domain Containing 3
TNF: Tumor Necrosis Factor
